# Author Correction: Non-toxic silver telluride colloidal quantum dot mid-infrared photodetector

**DOI:** 10.1038/s41467-026-78047-1

**Published:** 2026-09-23

**Authors:** So Young Eom, Jin Hyeok Lee, Haemin Song, Suheon Son, Kwang Seob Jeong

**Affiliations:** Department of Chemistry, Korea University, Seoul, Republic of Korea

**Keywords:** Quantum dots, Sensors and biosensors, Electronic materials, Mid-infrared photonics

Correction to: *Nature Communications* https://doi.org/10.1038/s41467-026-71374-3, published online 4 April 2026

In the version of the article initially published, ref. 33 was incorrect and has now been amended to “Guyot-Sionnest, P., Wehrenberg, B. & Yu, D. Intraband relaxation in CdSe nanocrystals and the strong influence of the surface ligands. *J. Chem. Phys*. **123**, 074709 (2005)” in the HTML and PDF versions of the article.

