# Supplementary material for: Non-toxic silver telluride colloidal quantum dot mid-infrared photodetector
Source: Nat Commun. 2026 Apr 4;17:4855. doi: 10.1038/s41467-026-71374-3 (PMC13223234; doi:10.1038/s41467-026-71374-3)
Supplement: Supplementary file 1 — Supplementary Information [file 41467_2026_71374_MOESM1_ESM.pdf]

Supplementary Information

Non-Toxic Silver Telluride Colloidal Quantum Dot Mid-Infrared Photodetector

So Young Eom[1,†], Jin Hyeok Lee[1,†], Haemin Song[1], Suheon Son[1], and Kwang Seob Jeong [1,*]

[1]Department of Chemistry, Korea University, Seoul 02841, Republic of Korea

[†]These authors contributed equally

[*]Corresponding author: kwangsjeong@korea.ac.kr

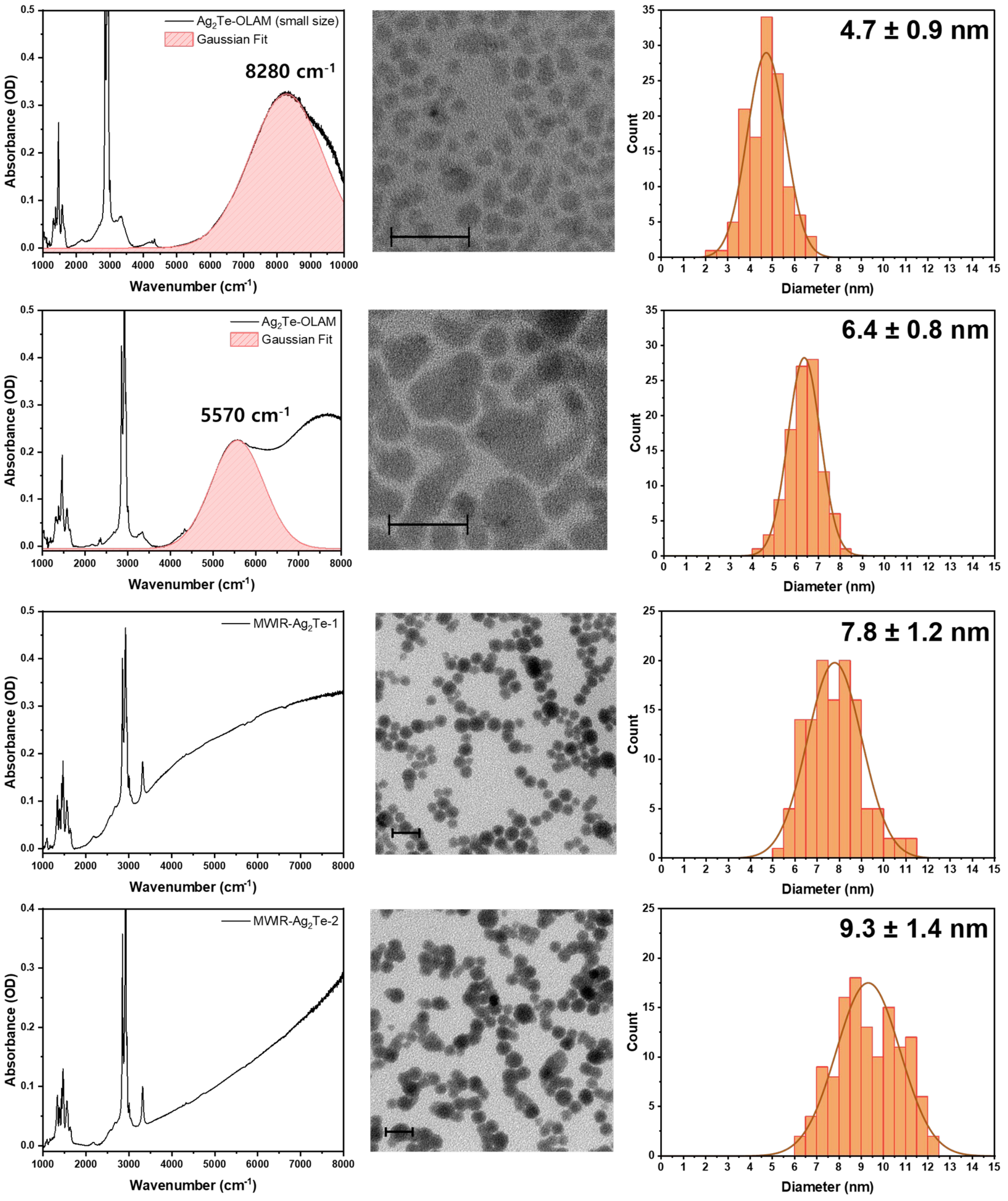

Supplementary Figure 1. Absorption spectrum, TEM image (scale bar = 20 nm), and size distribution of Ag_2Te (4~9 nm sizes) used for the 2-band k·p model.

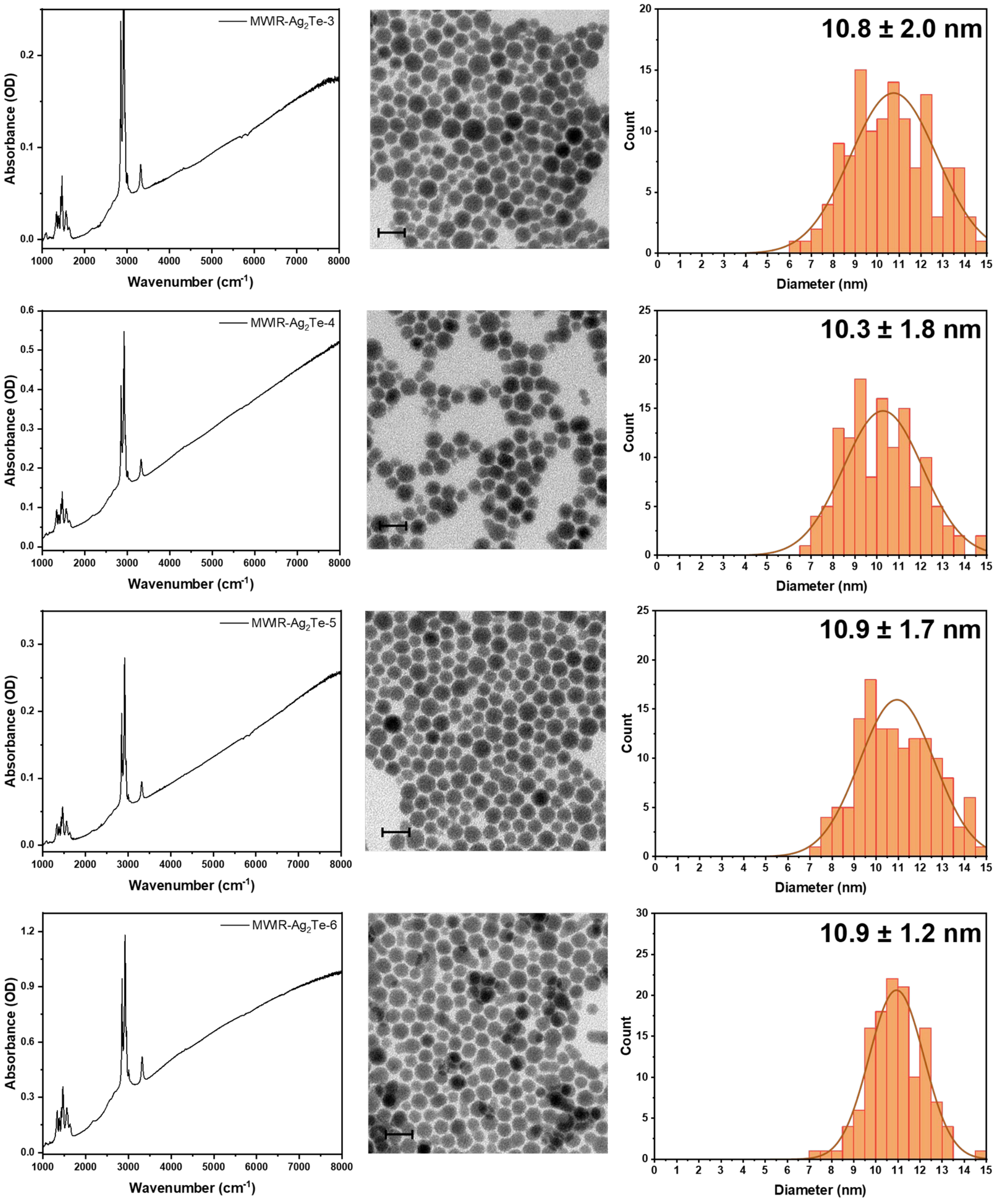

Supplementary Figure 2. Absorption spectrum, TEM image (scale bar = 20 nm), and size distribution of Ag_2Te (10~11 nm sizes) used for the 2-band k·p model.

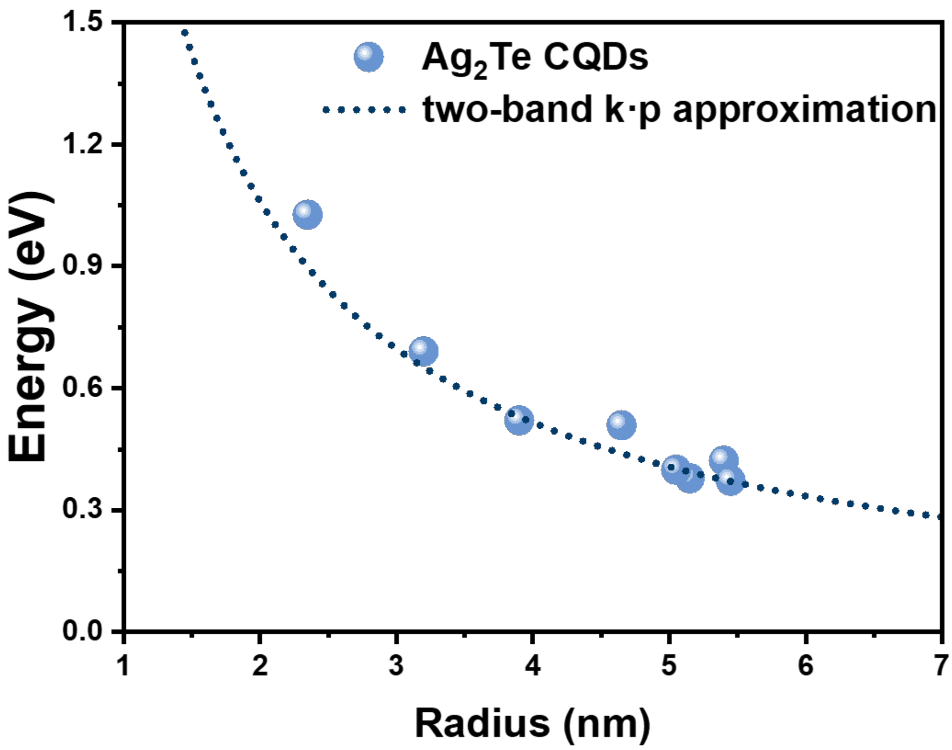

Supplementary Figure 3. Two-band k·p model of the bandgap energy as a function of radius, overlaid with experimental data[1].

$$E_g\ (QD) = -\frac{E_g\ (bulk)}{2} + \sqrt{\frac{Eg(bulk)^2}{4} + \frac{2}{3} \times \frac{\left(\frac{h}{2\pi}\right)^2 \times \left(\frac{\pi}{R(QD)}\right)^2}{2m_0}} \qquad (1)$$

The bulk bandgap of monoclinic β-Ag_2Te was taken as 0.06 eV, based on the experimentally reported value $E_0 = 0.064 \pm 0.009$ eV at 0 K[2]. As shown in Fig. 3, the particle sizes obtained from TEM measurement and their absorption spectra were analyzed. The Kane parameter (12 eV) was then calculated by fitting the experimental data to the model, aligning well with experimental data on how the transition energy changes with the radius of the QD.

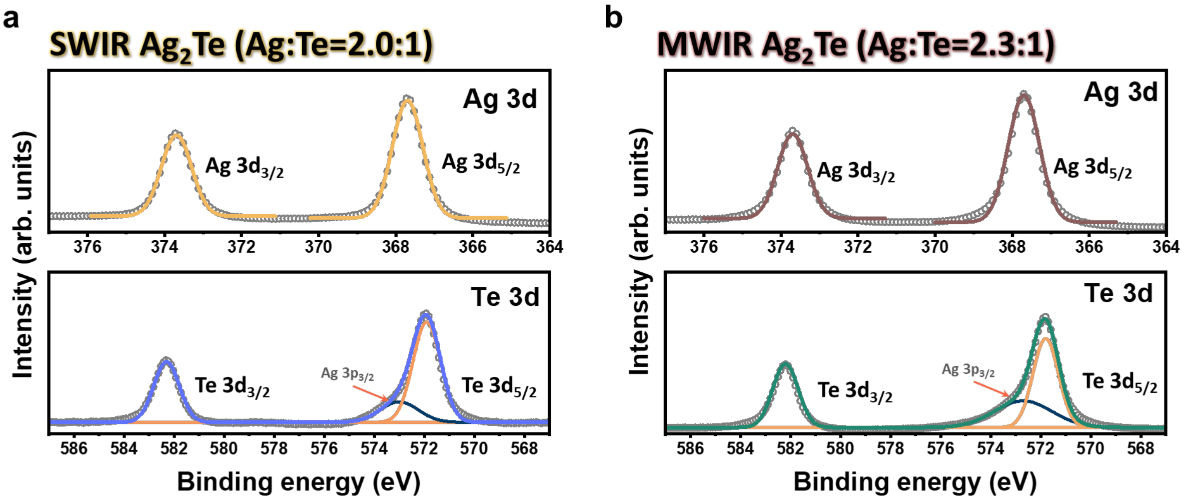

Supplementary Figure 4. XPS spectra a. Ag 3d and Te 3d core levels XPS spectra of SWIR Ag_2Te CQDs **b.** Ag 3d and Te 3d core levels XPS spectra of MWIR Ag_2Te CQDs synthesized via post-growth method.

XPS analysis shows that the Ag_2Te CQD composition is maintained when the CQDs further grow from SWIR CQD (2r= 6.3 nm, **a**) to the MWIR CQD (2r= 10.4 nm, **b**). The Ag $3d_{3/2}$, Ag $3d_{5/2}$, Te $3d_{3/2}$, Te $3d_{5/2}$ appear at 373.8 eV, 368.2 eV, 582.6 eV, and 572.0 eV, respectively. No discernible Ag(0) or Te-O is identified in the XPS spectra.

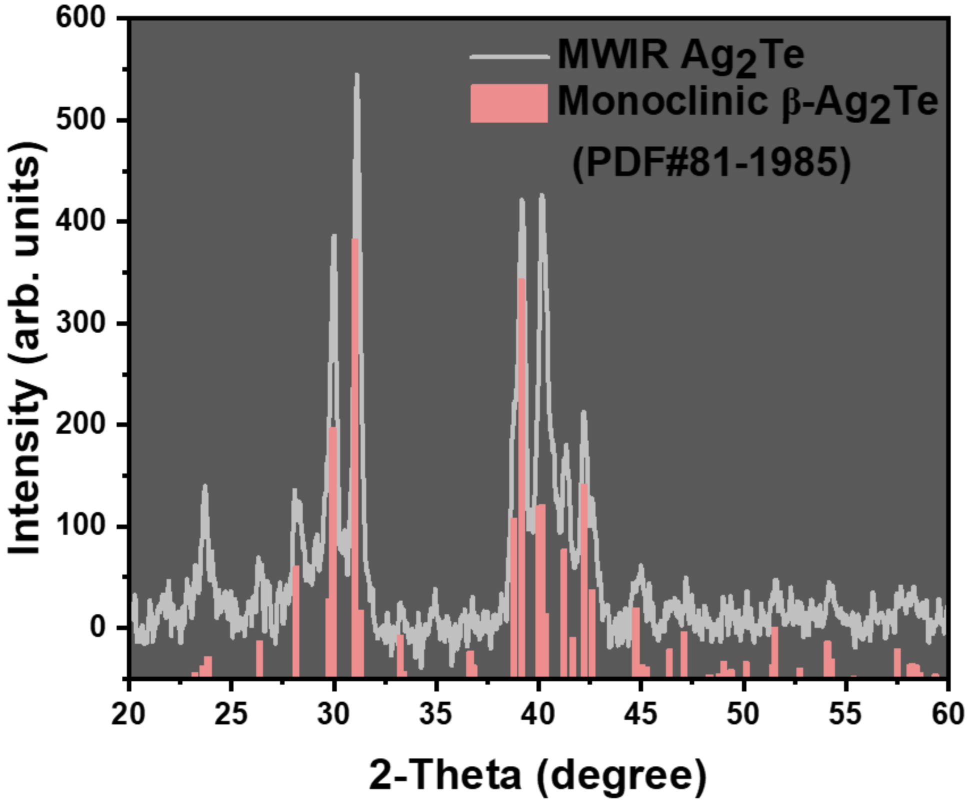

Supplementary Figure 5. XRD patterns of MWIR Ag_2Te CQDs compared with the bulk monoclinic β-Ag_2Te reference[3,4].

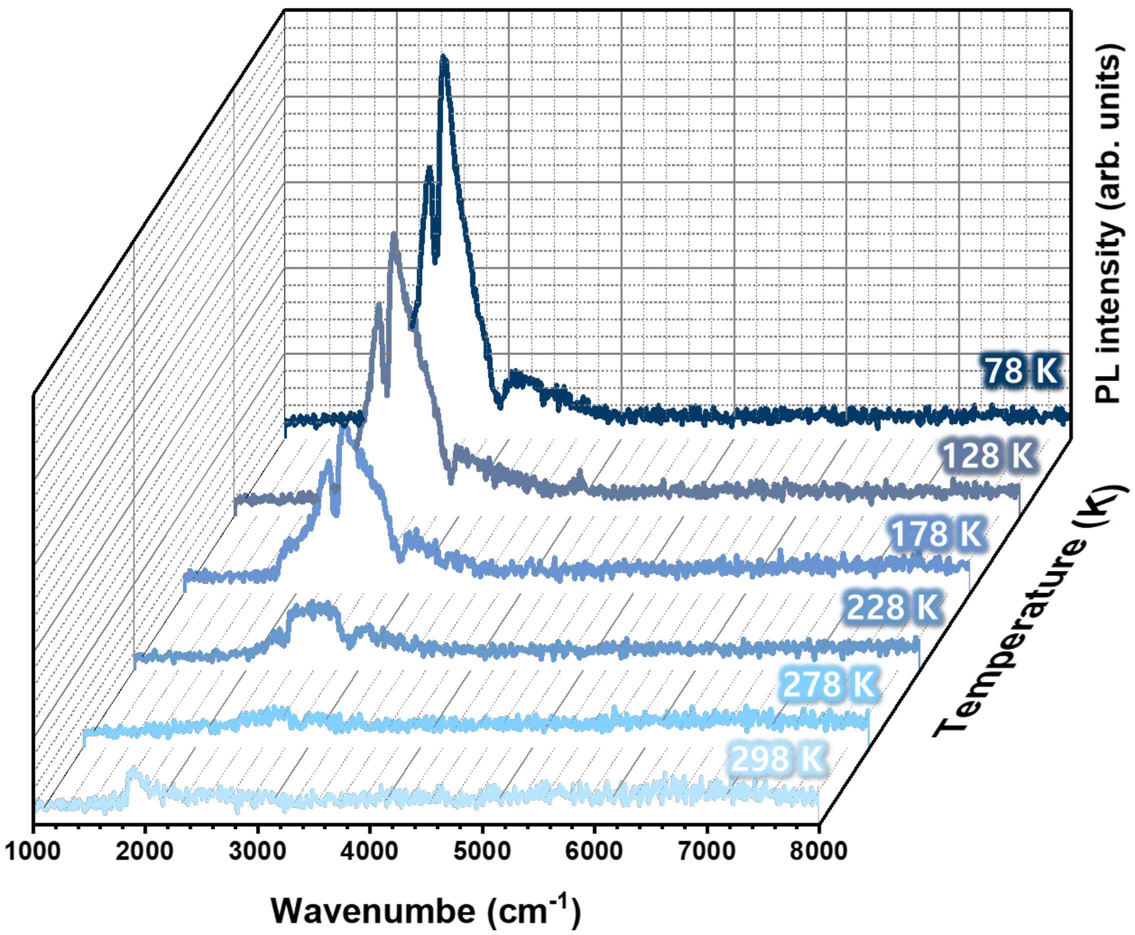

Supplementary Figure 6. Infrared photoluminescence spectra of MWIR Ag_2Te at various temperatures.

This behavior can be attributed to the correlation between the nonradiative relaxation time and temperature. The nonradiative relaxation time follows the relation:

$$k_{NR} = k_{EVET} + k_{AR} + k_{Tr} + \cdots \qquad (2)$$

The k_{NR}, k_{EVET}, k_{AR}, and k_{Tr} denotes the overall nonradiative relaxation rate, the electronic-to-vibrational energy transfer (EVET) rate, Auger recombination, and trap-assisted recombination processes, respectively. For the trap-assisted nonradiative recombination, carrier capture into trap states requires overcoming an activation energy barrier; therefore, the corresponding rate follows an Arrhenius-type temperature dependence. In the case of phonon-mediated nonradiative recombination, the phonon population follows the Bose–Einstein distribution, resulting in a temperature dependence[5].

$$n(\omega) = \frac{1}{e^{\left(\frac{\hbar\omega}{k_B T}\right)} - 1} \qquad (3)$$

where, $n(\omega)$, $\hbar, \omega, k_B$, and T are the average number of phonons, the reduced Planck constant, angular frequency, Boltzmann constant, and temperature, respectively. Consequently, both processes are suppressed at low temperatures, resulting in an increase in PL intensity.

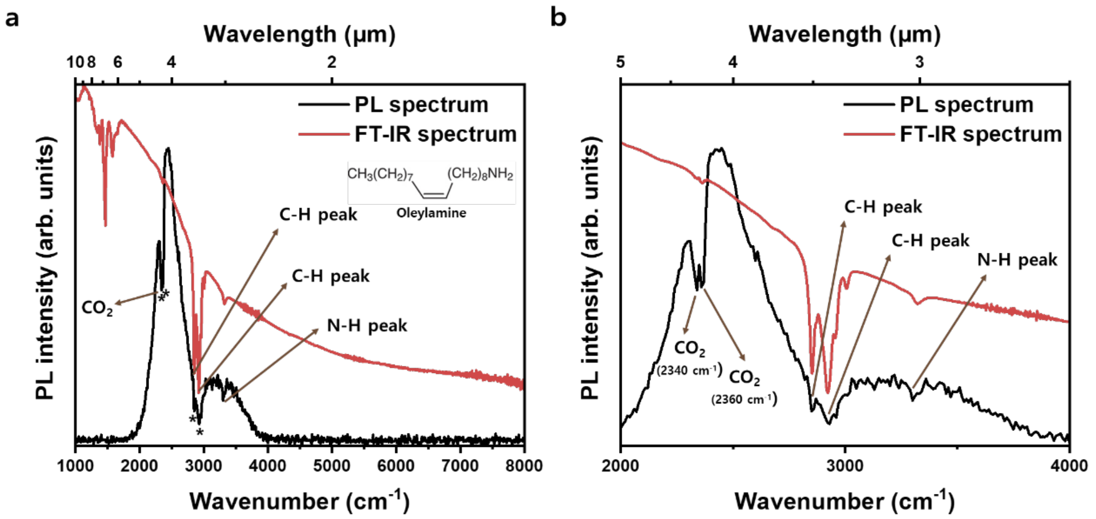

Supplementary Figure 7. Absorption and photoluminescence spectra of MWIR Ag_2Te. The absorbance spectra (b) provide an expanded view, highlighting the details of the vibrational energy peaks.

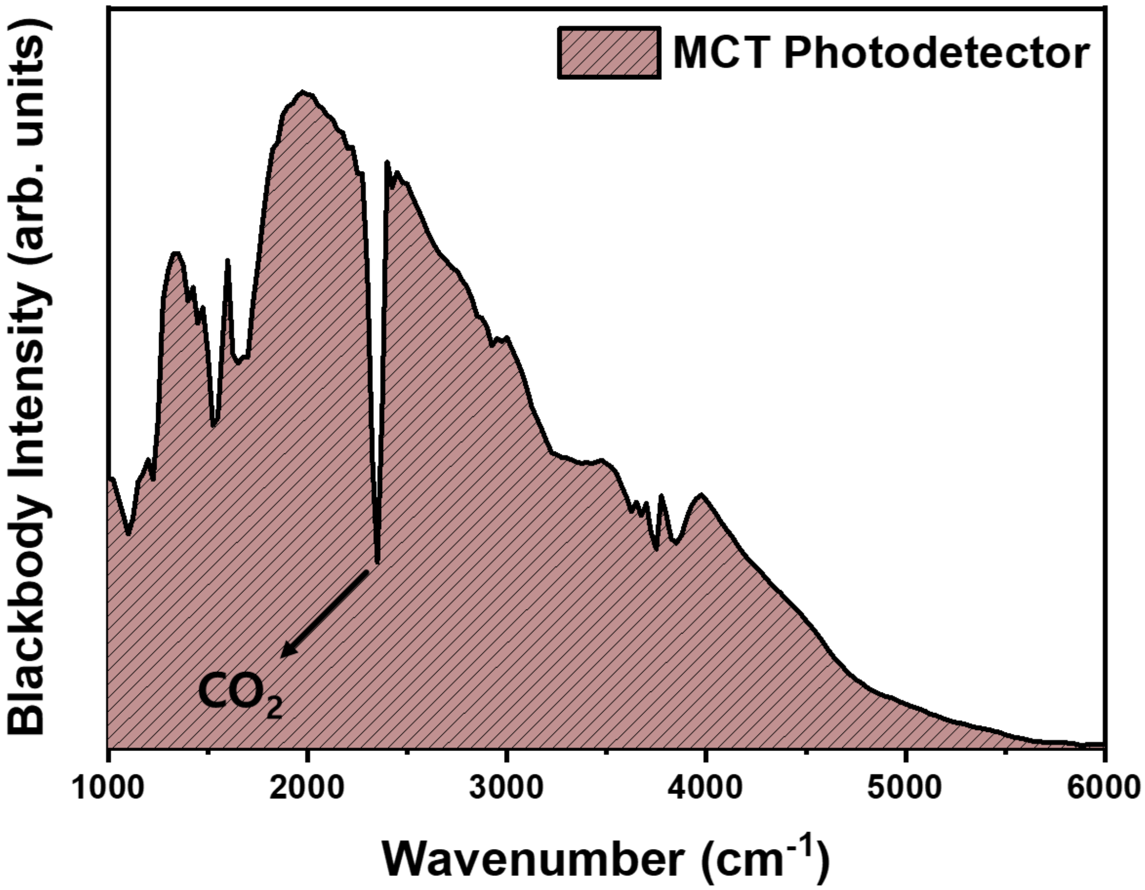

Supplementary Figure 8. Blackbody spectrum measured with a commercial MCT photodetector (I_{MCT}). This yields more detailed data in the 1000–6000 cm^{-1} range, as shown in Fig. 2b. The spectrum reveals the thermal radiation throughout the range and dips arising from the vibrational feature of CO_2 (2349 cm^{-1}) and water molecules in ambient conditions.

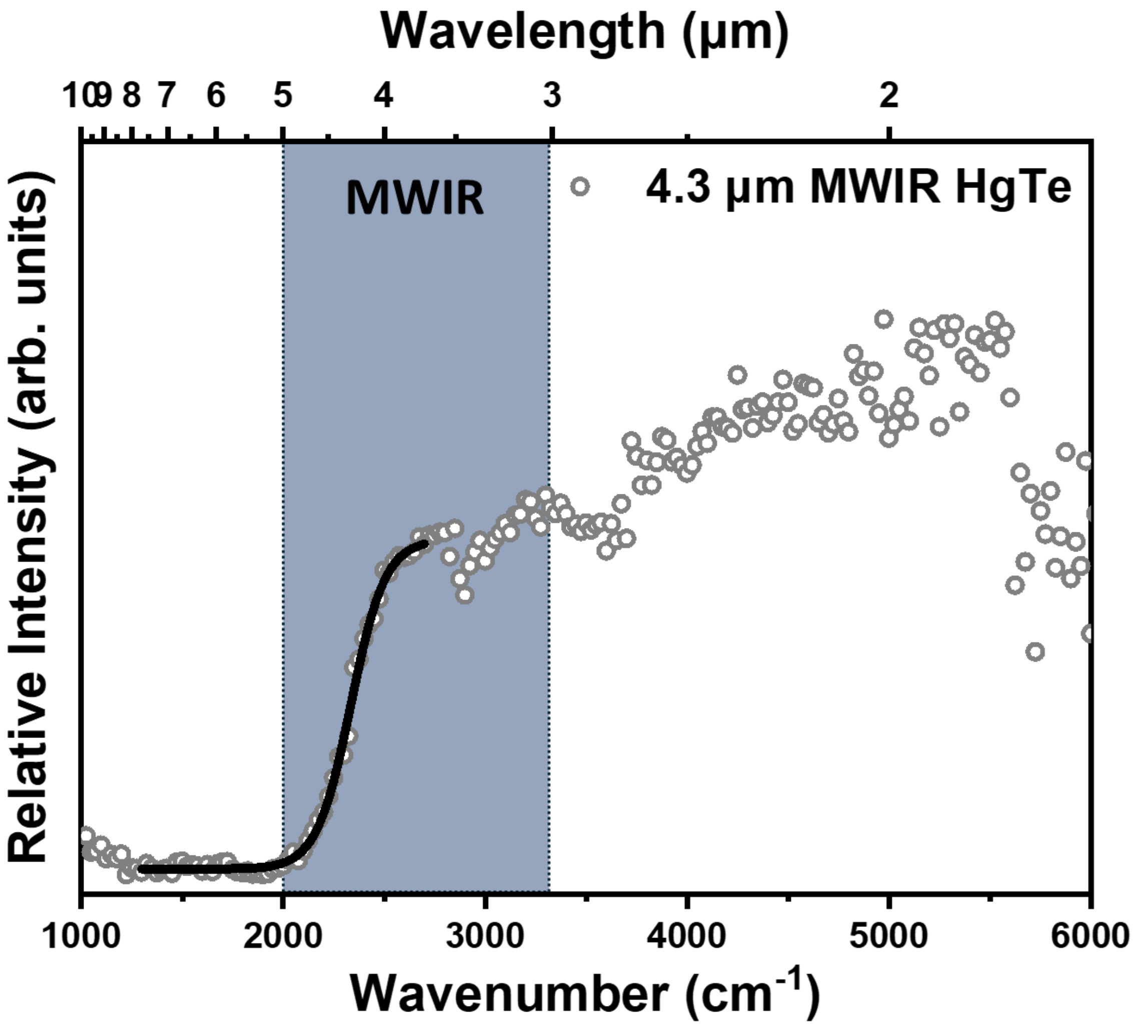

Supplementary Figure 9. Infrared photocurrent spectrum of MWIR HgTe CQDs. The synthesis procedure follows our previous report[6]. The CQDs, initially passivated with oleylamine and dodecanethiol ligands, go through the subsequently ligand-exchange with 1,2-ethanedithiol for film fabrication. The relative intensity was measured at 78 K.

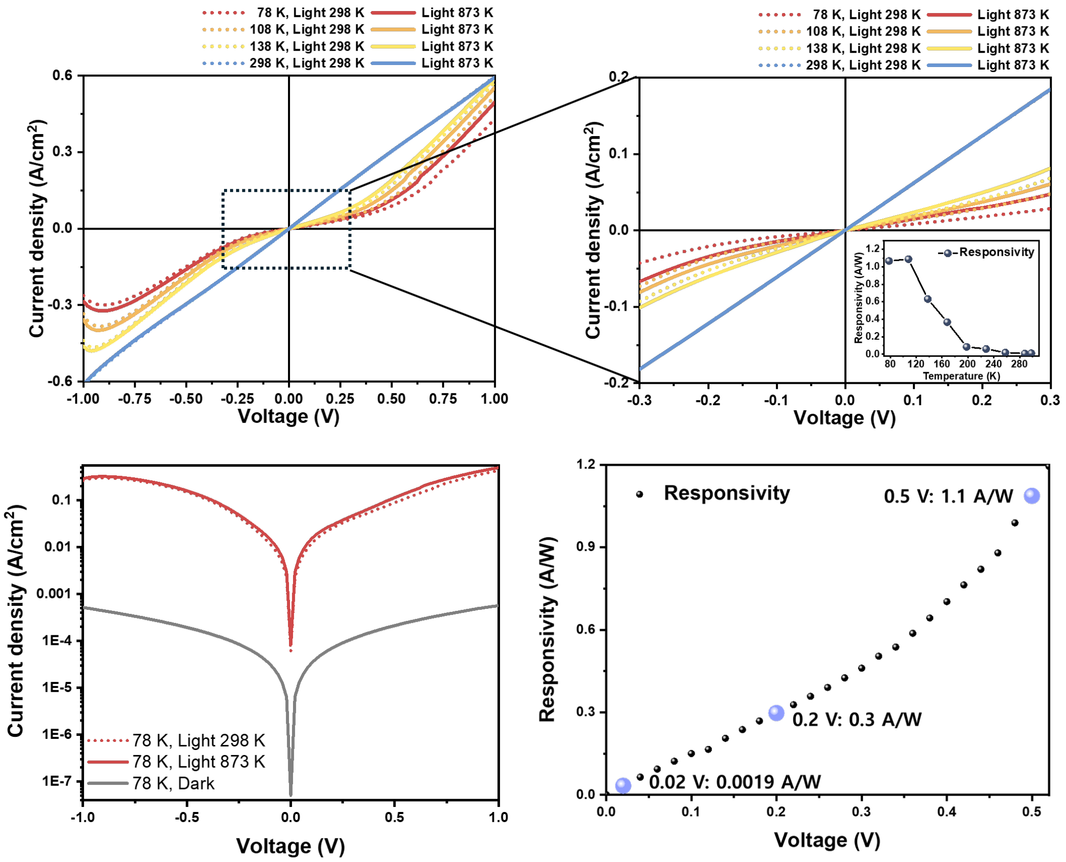

Supplementary Figure 10. Linear-scale J–V characteristics of the Ag_2Te CQD photoconductor measured under dark and illuminated conditions at different temperatures. The enlarged view around zero bias shows an approximately linear, symmetric response within $\pm$ 0.3 V. At the same time, a slight nonlinearity appears at higher bias, attributed to bulk-limited transport in the CQD film[7].

The responsivity was calculated using the following equation:

$$\text{Responsivity} = \left(\frac{I_{Light,873\ K} - I_{Light,298\ K}}{Light\ soucre\ power}\right) \qquad (4)$$

Specifically, the photocurrent is defined as the current difference between 873 K and 298 K conditions, and the responsivity is calculated using the incident blackbody radiation power at 873 K (28.1 mW/cm^2). The Light source power was determined to be 873 K blackbody power. At 0.5 V, the responsivity of 1.1 A/W was measured.

The J–V characteristics were measured under different illumination conditions (dark, 298 K, and 873 K), and the data were replotted on a logarithmic scale to more clearly highlight the differences between the dark and light-induced currents. The measured dark current density and light current density (873 K) are 5.1×10^{-8} A/cm^2 and 8.3×10^{-5} A/cm^2, respectively, yielding

an I_{Light}/I_{Dark} ratio of ~1.6×10^3. This confirms that the photoresponse is clearly distinguishable from the dark current. Additionally, the voltage–responsivity curve clearly shows that responsivity increases with increasing bias voltage.

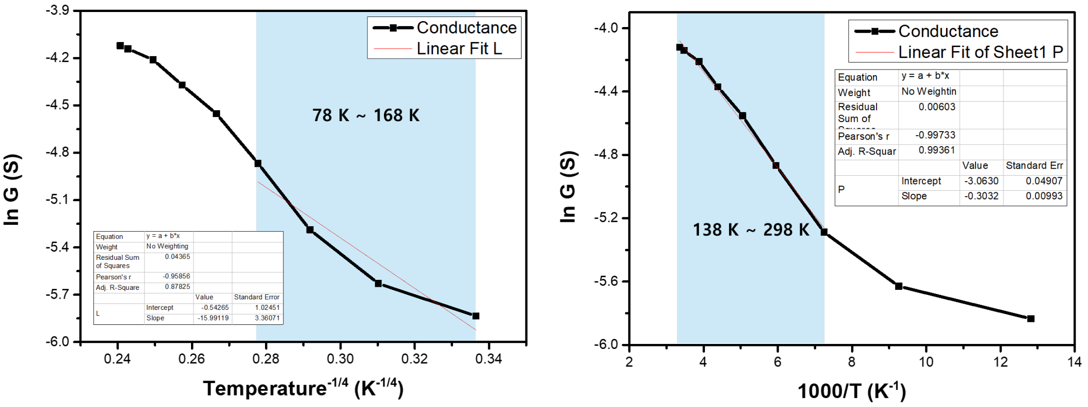

Supplementary Figure 11. Conductance (G) plotted against $T^{-1/4}$ and 1000/T, showing Mott behavior at low temperatures and Arrhenius behavior in the 138–168 K range[8,9].

A linear fit of $ln\, G$ versus $T^{-1/4}$ yields a slope equal to ${T_M}^{1/4}$, from which we obtain $T_M = 6.54 \times 10^4$. According to Mott's law, T_M can be expressed in the following equation:

$$T_m = \frac{\beta}{k_B g_0 \xi^3} \qquad (5)$$

The numerical constant β was set to 21.2. The g_0 is the density of states (DOS) at the Fermi level, and ξ is the localization length. Assuming ξ equals the size of QDs measured by TEM, the calculation yields the DOS value of $g_0 = 3.76 \times 10^{18}$, which is in the reasonable range for CQDs.

To examine whether the conductance transition follows Arrhenius behavior at higher temperatures, the data were plotted as follows.

$$G \propto \exp\left(-\frac{E_a}{k_b T}\right) \qquad (6)$$

The E_a, k_b are activation energy of carrier transport, Boltzmann constant. A linear fit of $ln\, G$ versus $1000/T$ yields a slope equal to $-E_a/1000k_b$.

$$\ln G = -3.0630 + (-0.3032) \times \frac{1000}{T} \qquad (7)$$

Accordingly, the activation energy was determined to be 26.1 meV. This value is similar to reference reports, confirming the reliability of the result and supporting the feasibility of thermally activated hopping. The temperature dependence shows a transition between variable-range hopping (VRH) and nearest-neighbor hopping (NNH). Using the two models, the

crossover temperature was calculated to be 145.5 K, suggesting that VRH dominates at lower temperatures, while NNH becomes prevalent at higher temperatures.

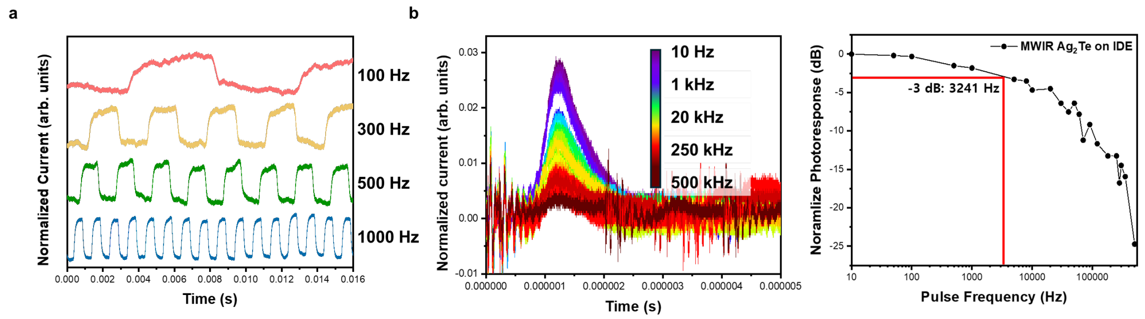

Supplementary Figure 12. Temporal and frequency-dependent photoresponse of the device. (a) Time-domain photoresponse measured under optical chopper modulation, showing distinct on–off switching behavior. (b) Pulse-frequency-dependent photoresponse measured by directly modulating the QCL pulse repetition frequency, illustrating the evolution of the response amplitude as a function of pulse frequency.

From the normalized frequency response, a −3 dB cutoff frequency of 3.2 kHz was extracted. This value is in good agreement with the cutoff frequency estimated from the measured fall time using the following relation:

$$\text{Fall-time and bandwidth relationship: } f_{3\,dB} \approx \frac{0.35}{\tau_{fall}} = 2.8\ kHz\ \ (\tau_{fall}: 124\ \mu s) \qquad (8)$$

The bandwidth derived from the pulse measurements is in good agreement with the data shown in Fig. 3d.

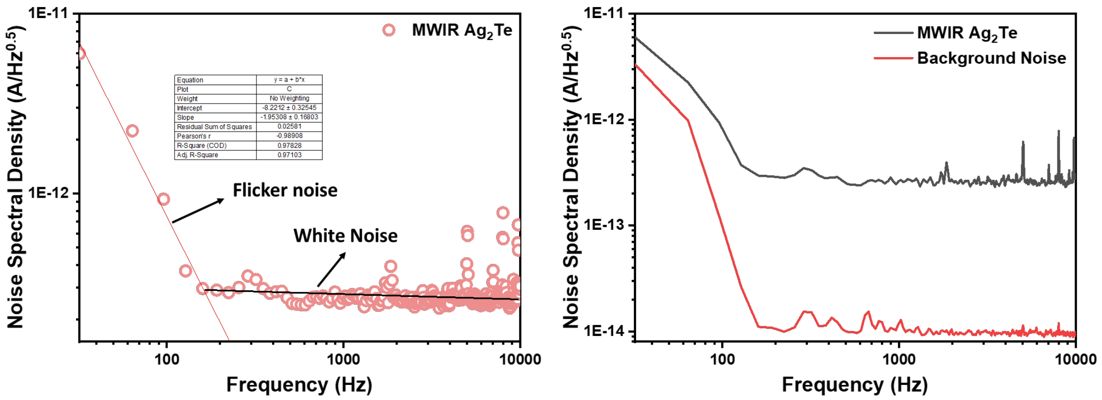

Supplementary Figure 13. The noise spectral density of the Ag_2Te photodetector was measured under dark conditions. At low frequencies (below ~100 Hz), the noise exhibits a characteristic 1/f dependence. As the frequency increases, the noise spectrum transitions to a frequency-independent region above ~100 Hz, corresponding to a white-noise-dominated regime.

Based on the dark current and the dynamic resistance measured at a bias of 0.02 V, the theoretical noise contributions were estimated.

The shot-noise-limited current noise: 0.23 $pA\sqrt{Hz}$, $i_{shot} = \sqrt{2qI\Delta f}$ (9)

The Johnson–Nyquist thermal noise: 0.19 $pA\sqrt{Hz}$,, $i_{theraml} = \sqrt{4k_B R\Delta f}$ (10)

The experimentally measured noise density of 0.26 $pA/Hz^{0.5}$ at 1 kHz shows close agreement with these theoretical limits. This correspondence indicates that at a modulation frequency of 1 kHz, low-frequency 1/f noise is effectively suppressed and the device operates near its intrinsic noise floor.

For comparison, the instrument noise floor of the measurement system was independently measured by shorting the input of the current preamplifier to ground. The resulting background noise level is significantly lower than the noise measured with the device across the entire frequency range of interest, including at 1 kHz. This confirms that the noise used for detectivity evaluation is dominated by the intrinsic device noise rather than by the measurement electronics.

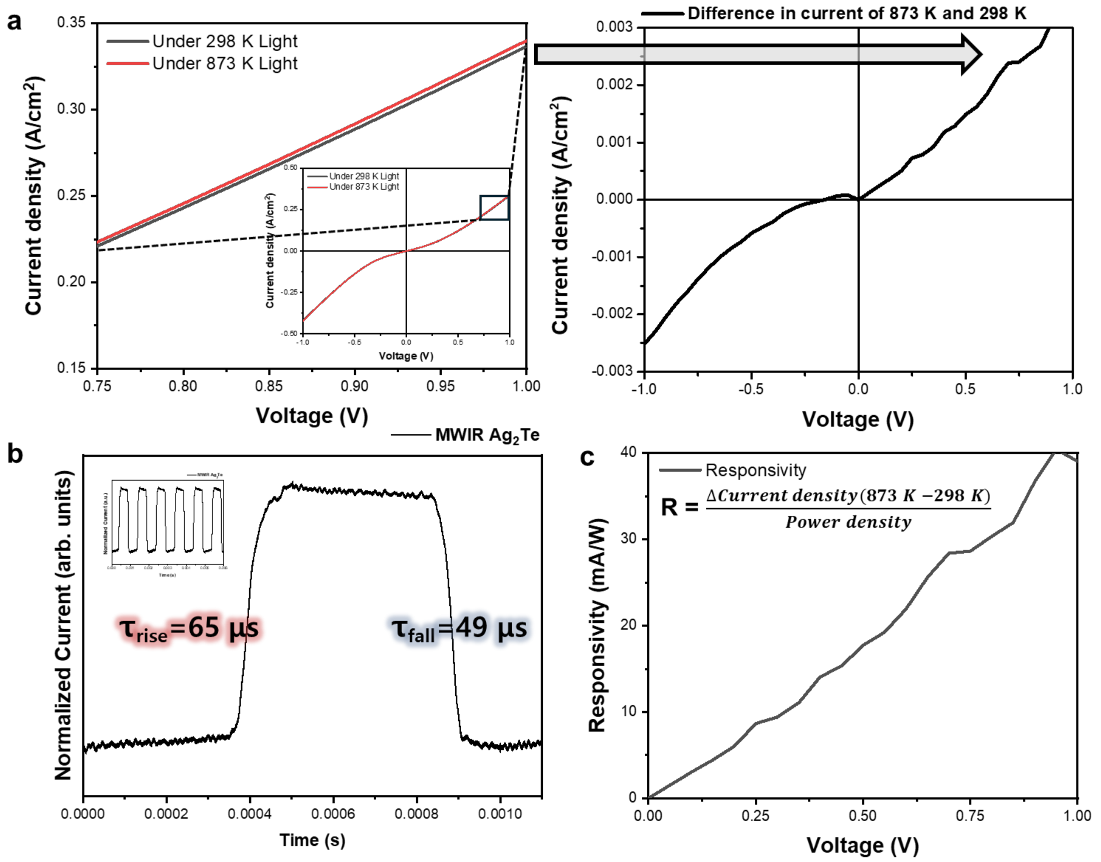

Supplementary Figure 14. (a) Current density–voltage (J-V) characteristics of the device measured in the dark and under illumination. The net photocurrent is shown, calculated as the difference between the two curves. (b) On–off photoresponse was measured using an optical chopper, while the intrinsic response time was evaluated using a pulsed laser because the chopper's modulation frequency was limited to 1 kHz. (c) Responsivity was calculated from current and power densities. The device's active area is 1.05 mm^2. At 1 V (positive bias), the responsivity is approximately 40 mA/W. The lower responsivity relative to the IDE structure is attributed to the low infrared transmittance of the ITO electrode at the MWIR regime.

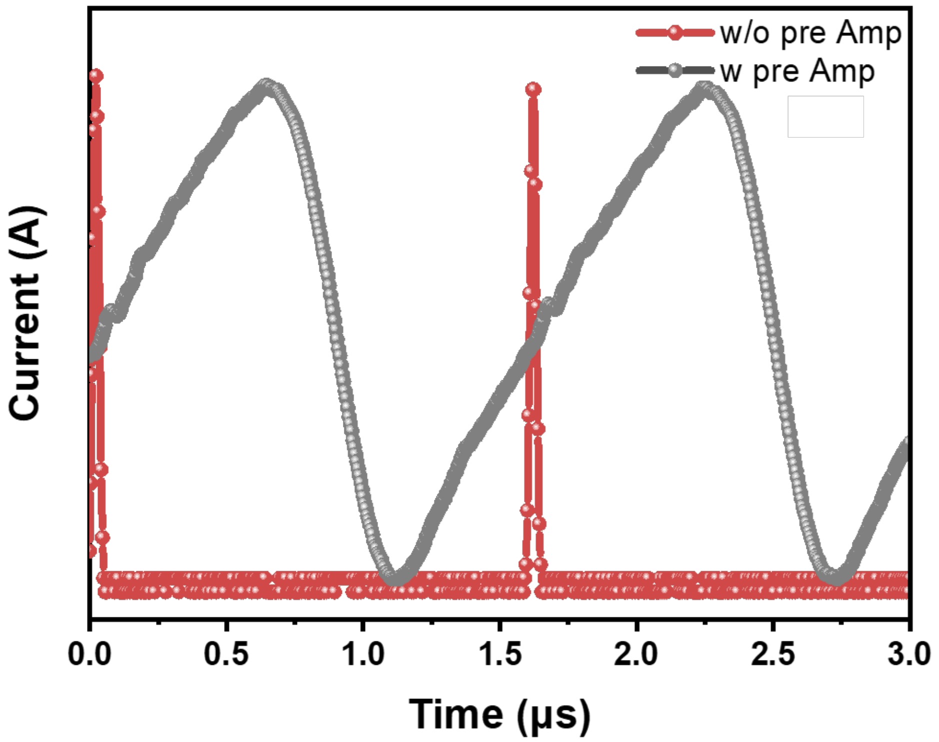

Supplementary Figure 15. The 625 kHz signal produced by a function generator was observed both with and without a preamplifier. The signal was either directly monitored with an oscilloscope (w/o Amp) or amplified by the preamplifier before recording (w/ Amp). The results confirm the presence of an instrument response function (IRF).

Supplementary Table 1. Performance of the detectivity table of non-toxic MWIR and SWIR Ag_2Te photodetectors from previous studies.

Materials	Detectivity	Wavelength	Year	Ref
**InSb**	4.7×10^7 Jones<br>3.1×10^7 Jones	3.0 µm<br>3.5 µm	2025	10
**SnTe**	N/A	3.0 µm	2018	11
**Ag_2Se**	7.8×10^6 Jones	4.5 µm<br>(Intraband)	2021	12
**Ag_2Te**	3.0×10^{12} Jones	1.3 µm	2024	13
	9.0×10^{10} Jones	1.7 µm	2024	14
	1.0×10^7 Jones	2.0 µm	2024	15
	**1.2×10^9 Jones**	**4.7 µm**	**2026**	**This Work**

Supplementary Table 2. Performance table of SWIR/MWIR photodetectors from previous studies

Photoactive materials	Cut-off [μm]	Fall time [μs]	Device type	Temperature [K]	Year	Ref
**Black phosphorus**	4	2.5	PC	298	2025	16
**Ag_2Te**	1.4	3.3	PD	298	2024	13
**Ag_2Te**	1.7	2.7	PD	298	2024	14
**InSb**	4.4	2790	PC	298	2025	10
**HgTe**	4	11	PD	80	2022	17
**HgSe/HgTe**	5 (Intraband)	0.5	PD	80	2019	18
**HgTe**	3.5 ~ 4	5	PD	80	2021	19
**HgTe**	5 (Dual band)	2.5	PD	85	2019	20
**HgTe**	2.3	8.9	PD	298	2023	21
**HgTe**	5 (Plasmonic resonance)	0.06	PC	298	2024	22
**Ag_2Te**	**4.7**	**0.523**	**PC**	**78**	**2026**	**This work**

Supplementary References

1. Lhuillier, E., Keuleyan, S. & Guyot-Sionnest, P. Optical properties of HgTe colloidal quantum dots. *Nanotechnology* **23**, 175705 (2012).
2. Dalven, R. & Gill, R. Energy Gap in β − Ag 2 Te. *Phys. Rev.* **143**, 666–670 (1966).
3. Sahu, A., Qi, L., Kang, M. S., Deng, D. & Norris, D. J. Facile Synthesis of Silver Chalcogenide (Ag_2 E; E = Se, S, Te) Semiconductor Nanocrystals. *J. Am. Chem. Soc.* **133**, 6509–6512 (2011).
4. Kumar, N., Sinha Ray, S. & Ngila, J. C. Ionic liquid-assisted synthesis of Ag/Ag_2 Te nanocrystals *via* a hydrothermal route for enhanced photocatalytic performance. *New J. Chem.* **41**, 14618–14626 (2017).
5. Antonius, G. & Louie, S. G. Theory of exciton-phonon coupling. *Phys. Rev. B* **105**, 085111 (2022).
6. Song, H. *et al.* Narrow bandgap silver mercury telluride alloy semiconductor nanocrystal for self-powered midwavelength-infrared photodiode. *Commun Mater* **5**, 60 (2024).
7. Strasfeld, D. B., Dorn, A., Wanger, D. D. & Bawendi, M. G. Imaging Schottky Barriers and Ohmic Contacts in PbS Quantum Dot Devices. *Nano Lett.* **12**, 569–575 (2012).
8. Kang, M. S., Sahu, A., Norris, D. J. & Frisbie, C. D. Size- and Temperature-Dependent Charge Transport in PbSe Nanocrystal Thin Films. *Nano Lett.* **11**, 3887–3892 (2011).
9. Liu, H., Pourret, A. & Guyot-Sionnest, P. Mott and Efros-Shklovskii Variable Range Hopping in CdSe Quantum Dots Films. *ACS Nano* **4**, 5211–5216 (2010).
10. Liu, Z. *et al.* Colloidal InSb Quantum Dots Mid-Wave Infrared Photoconductive Detectors via One-Step Strong Acid Surface Treatment Strategy. *Nano Lett.* **25**, 13549–13556 (2025).
11. Cryer, M. E. & Halpert, J. E. Room Temperature Mid-IR Detection through Localized Surface Vibrational States of SnTe Nanocrystals. *ACS Sens.* **3**, 2087–2094 (2018).
12. Hafiz, S. B., Al Mahfuz, M. M. & Ko, D.-K. Vertically Stacked Intraband Quantum Dot Devices for Mid-Wavelength Infrared Photodetection. *ACS Appl. Mater. Interfaces* **13**, 937–943 (2021).
13. Wang, Y. *et al.* Silver telluride colloidal quantum dot infrared photodetectors and image sensors.

Nat. Photon. **18**, 236–242 (2024).

14. Kim, G. *et al.* Extended Short-Wavelength Infrared Ink by Surface-Tuned Silver Telluride Colloidal Quantum Dots and Their Infrared Photodetection. *ACS Materials Lett.* **6**, 4988–4996 (2024).
15. Ahn, Y. *et al.* Silver Telluride Colloidal Quantum Dot Solid for Fast Extended Shortwave Infrared Photodetector. *Advanced Science* **11**, 2407453 (2024).
16. Wijaya, T. J. *et al.* Mechanically flexible mid-wave infrared imagers using black phosphorus ink films. *Nat Commun* **16**, 5972 (2025).
17. Dang, T. H. *et al.* Broadband Enhancement of Mid-Wave Infrared Absorption in a Multi-Resonant Nanocrystal-Based Device. *Advanced Optical Materials* **10**, 2200297 (2022).
18. Livache, C. *et al.* A colloidal quantum dot infrared photodetector and its use for intraband detection. *Nat Commun* **10**, 2125 (2019).
19. Dang, T. H. *et al.* Bias Tunable Spectral Response of Nanocrystal Array in a Plasmonic Cavity. *Nano Lett.* **21**, 6671–6677 (2021).
20. Tang, X., Ackerman, M. M., Chen, M. & Guyot-Sionnest, P. Dual-band infrared imaging using stacked colloidal quantum dot photodiodes. *Nat. Photonics* **13**, 277–282 (2019).
21. Yang, J. *et al.* Bi_2S_3 Electron Transport Layer Incorporation for High-Performance Heterostructure HgTe Colloidal Quantum Dot Infrared Photodetectors. *ACS Photonics* **10**, 2226–2233 (2023).
22. Caillas, A. & Guyot-Sionnest, P. Uncooled High Detectivity Mid-Infrared Photoconductor Using HgTe Quantum Dots and Nanoantennas. *ACS Nano* **18**, 8952–8960 (2024).
